# CGG Repeats in the 5’UTR of FMR1 RNA Regulate Translation of Other RNAs Localized in the Same RNA Granules

**DOI:** 10.1371/journal.pone.0168204

**Published:** 2016-12-22

**Authors:** René Rovozzo, George Korza, Mei W. Baker, Meng Li, Anita Bhattacharyya, Elisa Barbarese, John H. Carson

**Affiliations:** 1 Center for Cell Analysis and Modeling, UConn Health, Farmington, Connecticut, United States of America; 2 Department of Molecular Biology and Biophysics, UConn Health, Farmington, Connecticut, United States of America; 3 Departments of Pediatrics, School of Medicine and Public Health, University of Wisconsin-Madison, Wisconsin State Laboratory of Hygiene, Newborn Screening Laboratory, Madison, Wisconsin, United States of America; 4 Waisman Center, University of Wisconsin, Madison, Wisconsin, United States of America; 5 Department of Neuroscience, UConn Health, Farmington, Connecticut, United States of America; Centre National de la Recherche Scientifique, FRANCE

## Abstract

CGG repeats in the 5’UTR of Fragile X Mental Retardation 1 (FMR1) RNA mediate RNA localization and translation in granules. Large expansions of CGG repeats (> 200 repeats) in FMR1, referred to as full mutations, are associated with fragile X syndrome (FXS). Smaller expansions (55–200 repeats), referred to as premutations, are associated with fragile X tremor ataxia syndrome (FXTAS) and fragile X premature ovarian insufficiency (FXPOI). TMPyP4 is a porphyrin ring compound that destabilizes CGG repeat RNA secondary structure. Here we show that exogenous CGG repeat RNA by itself, lacking the FMRP ORF, microinjected into hippocampal neurons is localized in RNA granules and inhibits translation of ARC RNA, which is localized in the same granules. TMPyP4 rescues translation of ARC RNA in granules. We also show that in human premutation fibroblasts with endogenous CGG repeat expansions in the FMR1 gene, translation of ARC RNA is inhibited and calcium homeostasis is disrupted and both phenotypes are rescued by TMPyP4. Inhibition of granule translation by expanded CGG repeats and rescue of granule translation by TMPy4, represent potential pathogenic mechanism and therapeutic strategy, respectively, for FXTAS and FXPOI.

## Introduction

CGG repeat sequences have been identified in >200 different RNAs in the human exome [1]. In some cases expansion of CGG repeats is associated with neurological or neuromuscular disorders [2]. For example, the Fragile X Mental Retardation 1 (FMR1) gene, encoding fragile X mental retardation protein (FMRP), normally contains 5–55 CGG repeats in the 5’UTR. Large expansions of CGG repeats (> 200 repeats) in the FMR1 gene, referred to as full mutations, cause DNA methylation and transcriptional silencing, resulting in fragile X syndrome (FXS), a neurodevelopmental disorder characterized by intellectual disability and autism [3]. Smaller expansions of CGG repeats (55–200 repeats) in the same gene, referred to as premutations, are associated with fragile X tremor ataxia syndrome (FXTAS), a late onset neurodegenerative disorder characterized by tremor, ataxia and cognitive decline [4–6] and fragile X premature ovarian insufficiency (FXPOI), characterized by infertility and early menopause [7].

Most eukaryotic RNAs undergo conventional translation, which initiates at an AUG start codon at the beginning of the open reading frame (ORF) and terminates at a stop codon at the end of the ORF, resulting in synthesis of the protein encoded by the ORF. RNAs such as FMR1, which contain trinucleotide repeats in the 5’UTR, can also undergo an unconventional type of translation, called repeat associated non-AUG (RAN) translation, which initiates at non-AUG sites in the vicinity of the repeats in the 5’UTR, resulting in synthesis of poly-amino acid RAN translation products encoded by the repeat sequences [8–11]. Pathogenesis of FXTAS is believed to reflect toxicity of either the CGG repeat expansion RNA itself or of RAN translation products encoded by CGG repeat expansion RNA, although the mechanism(s) of toxicity are unclear.

CGG repeats can form secondary structures (hairpins, duplexes) by a combination of canonical C(anti)::G(anti) and non-canonical G(syn)::G(anti) base pairing [12], which may cause ribosomes to stall in the CGG repeat region of the 5’UTR. Expanded CGG repeats in FMR1 RNA are associated with reduced translation of the downstream FMRP ORF [13,14], suggesting that expanded CGG repeats might increase stalling of ribosomes in the 5’UTR, decreasing translation of the downstream ORF. TMPyP4 (tetra-(N-methyl-4-pyridyl) porphyrin) is a membrane-permeant porphyrin ring compound that binds to CGG repeat RNA and destabilizes RNA secondary structure. TMPyP4 reverses the effect of expanded CGG repeats on FMRP translation [13,14], possibly by preventing ribosome stalling in the CGG repeat region.

Heterogeneous nuclear ribonucleoprotein (hnRNP) A2 binds to CGG repeats, which are found in multiple different RNAs, including FMR1 RNA [15–17], and also to A2 response elements (A2RE), which are also found in multiple RNAs, including activity regulated cytoskeletal associated protein (ARC) RNA [18]. Single molecule imaging reveals that both FMR1 RNA and ARC RNA are localized and translated in granules and that newly-synthesized FMRP and ARC protein molecules both accumulate in the vicinity of the granules where they are synthesized [19].

Since FMR1 RNA, and ARC RNA, are both localized and translated in the same RNA granules [19], and since expanded CGG repeats in FMR1 RNA inhibit translation of FMRP, possibly by causing ribosomes to stall in the 5’UTR, the presence of expanded CGG repeat RNA in granules might affect conventional translation of ARC RNA localized in the same granules and TMPyP4 might block this effect. In this regard, expression of CGG repeat expansion RNA in transgenic mice and flies does affect translation of other RNAs [20].

Several RNAs containing CGG repeats or A2RE sequences, which may be localized in the same granules as FMR1 RNA, encode proteins that regulate calcium homeostasis in the cell [1,18]. Previous work has shown that calcium transients are increased in astrocytes from CGG KI mice [21] and in iPSC-derived neurons from individuals with FXTAS [22], both of which contain CGG repeat expansions. If CGG repeat expansions in FMR1 RNA affect translation of other RNAs in the same granule this could potentially affect calcium homeostasis in the cell.

Here we show that exogenous CGG repeat RNA, microinjected into neurons and endogenous CGG repeat expansions in FMR1 RNA expressed in premutation fibroblasts both inhibit translation of ARC RNA, which is localized in the same granules as FMR1 RNA and serves as a reporter for translation in granules, and that calcium homeostasis is also affected in premutation fibroblasts. Furthermore, TMPyP4 rescues translation of ARC RNA in neurons and in premutation fibroblasts and also rescues calcium homeostasis in premutation fibroblasts.

## Materials and Methods

### Ethics statement

Use of animals as described in this study followed the guidelines of UConn Health and those of the National Institutes of Health for the Care and Use of Laboratory Animals, and was approved under Protocol # 100686–0416 by the Institutional Animal Care and use Committee (IACUC) of UConn Health. UConn Health complies with all applicable provisions of the Animal Welfare Act and other Federal statutes and regulations relating to animals. UConn Health Animal Welfare Assurance number is A3471-01.

Skin biopsies were obtained from selected patients at the Waisman Center at the University of Wisconsin—Madison following recruitment, consent and sample collection procedures approved by the University of Wisconsin-Madison Health Sciences Institutional Research Board of the Human Research Protection Program (HRPP). Prior to consent and sample collection potential subjects were screened in person to determine study eligibility. Subjects found to be eligible underwent approved consent procedures, including written informed consent. Skin biopsies were obtained by a physician at the Waisman Center and fibroblasts were isolated from skin biopsies.

The University of Connecticut Health Institutional Review Board has specifically exempted the use of human cell lines (fibroblasts produced at the Waisman Center at the University of Wisconsin-Madison and fibroblasts obtained from the Coriell Institute for Medical Research) in this study from IRB approval because the cell lines are de-identified.

### CGG repeat profiles

Profiles of sequential CGG repeats in target sequences were calculated using a sliding sequence algorithm that compares a query sequence consisting of four sequential CGG repeats to sequential consecutive overlapping 12 nt sequences in the target RNA sequence and calculates homology scores at each position. Perfect homology between the query sequence and the target sequence yields a homology score of 1 at that position. Homology scores < 1 indicate less than perfect homology. Multiple sequential CGG repeats in the target sequence produce a profile of peaks with homology scores of 1 at positions where the CGG repeats in the query and target sequences are aligned, separated by intervening lower scores where the CGG repeats in the query and target sequences are misaligned.

### Ribosome profiles

Ribosome profiling is a technique that reveals the probability of ribosomes localized at each position on an RNA molecule [23]. In most RNAs, ribosome probability is high in the ORF of the RNA, reflecting active translation in this region, and low in the 5’UTR and 3’UTR, indicating less active translation in these regions. In RNAs with trinucleotide repeats or upstream ORFs in the 5’UTR, ribosome probability in the 5’UTR may be increased if translation is increased in these regions. However, ribosome probability in regions of CGG repeats may be underestimated by ribosome profilng because sequencing through long regions of CGG repeats is inefficient [24]. Ribosome profiles for FMR1 RNA, representing global aggregate data from multiple published studies with multiple different cell types under multiple different conditions [25–35], were obtained from the GWIPS-viz database (http://gwips.ucc.ie).

### In vitro transcription

ARC RNA and Venus-ARC RNA were prepared by in vitro transcription of linearized plasmid DNA, as previously described [18,19]. CGG 0, 30, 62 and 99 RNAs were prepared by in vitro transcription from plasmid DNA containing FMR1 cDNA with 0, 30, 62 or 99 CGG repeats, respectively, obtained from Dr. Fry [13]. Plasmid DNAs were linearized at the Xho 1 site immediately downstream of the CGG repeat region. Labeled RNAs were prepared by in vitro transcription in the presence of Cy3- or Cy5-conjugated UTP (all UTPs from GE Healthcare Biosciences, Pittsburgh, PA) using T7 mScript Standard mRNA Production System, according to manufacturer's protocol (CellScript, Madison, WI) or Amplicap-Max T3 High Yield Message Maker Kit (CellScript, Madison, WI) followed by capping and polyadenylation using reagents supplied in the T7 mScript Standard mRNA Production System (CellScript, Madison, WI) or Amplicap-Max T3 High Yield Message Maker Kit (CellScript, Madison, WI). Intactness and purity of in vitro transcribed RNAs were analyzed by gel electrophoresis and fluorescence correlation spectroscopy (FCS).

### Surface plasmon resonance analysis

Biotin labeled CGG 0, 30, 62 and 99 RNA ligands prepared by in vitro transcription in the presence of biotin-UTP, were immobilized on a streptavidin Biacore chip, and binding to serial dilutions of TMPyP4 analyte was measured using a Biacore T100 instrument. Sensorgrams were fitted to a heterogenous ligand model to determine binding parameters for specific and non-specific binding of TMPyP4 to each RNA.

### Cell culture, microinjection and confocal imaging

Hippocampal neurons obtained from C57Bl/6 E19 mouse embryos were grown in culture as described previously [18]. Human fibroblasts were obtained from unaffected normal controls with normal numbers of CGG repeats in the FMR1 gene and from apparently unaffected female premutation carriers with expanded numbers of CGG repeats in the FMR1 gene (see “Human fibroblasts” below). Fibroblasts were grown in culture as recommended by the Coriell Institute cell repository with the addition of alpha-tocopherol (10 micrograms/ml) (Sigma Cat# T3251-5G), a lipophilic radical scavenger used to reduce cellular autofluorescence in cultured human fibroblast cells [36].

Primary mouse hippocampal neurons (14 to 21 days in culture) or human fibroblasts, grown in the presence or absence of 1 micromolar TMPyP4 (Calbiochem, Darmstadt, Germany) were microinjected using an electronic microinjection system (Eppendorf, Hamburg, Germany). Injected cells were identified by fluorescence microscopy. Three channel confocal images of injected cells were collected by recording simultaneously in the 488 nm, 560 nm and 633 nm channels using a Zeiss LSM 510 confocal laser scanning microscope with a 63X 1.4 numerical aperture oil immersion objective lens (Carl Zeiss, Thornwood, NY). Images were collected with maximum dynamic range and minimum background in each channel, while avoiding image saturation (pixels >255) or image offset below background (pixels <0). Individual RNA granules were masked and fluorescent intensity for each granule was integrated in each channel after subtracting background fluorescence using Image J. Specific translational activities were calculated by dividing the fluorescence intensity of newly-synthesized Venus-ARC protein, measured in the 488 nm channel, by the fluorescence intensity of Venus-ARC RNA, measured in the 633 nm channel.

### Fluorescence Correlation Spectroscopy (FCS)

The numbers of fluorescent RNA molecules in individual granules were determined by FCS [37,38]. Counts per molecule for Cy5-conjugated-UTP-labelled CGG 99 RNA and Alexafluor 488-conjugated-UTP-labelled ARC RNA were first determined by FCS in solution. Both RNAs were co-injected into primary mouse hippocampal neurons in culture. Dual channel confocal images of injected cells were collected to visualize individual granules containing both fluorophores. The FCS observation volume was positioned to encompass a single RNA granule and FCS counts were recorded for 10 s, during which the count rate decayed due to photobleaching of fluorescent RNA molecules in the granule, which is immobile. The numbers of fluorescent RNA molecules of each type in each granule were determined by subtracting counts remaining after 10 s from counts at time 0 and dividing by counts per molecule.

### Calcium transients

Fibroblasts in culture were loaded with Fluo-4 calcium indicator, according to the manufacturer’s protocol (Life Technologies). Bradykinin was bath applied to the cells to induce calcium transients. Fluo-4 fluorescence was recorded by time lapse confocal microscopy after bradykinin addition. Fluo-4 fluorescence in individual cells was quantified by masking each cell and integrating total fluorescence in the cell at each time step. Calcium transients were expressed as F/F_0_.

### Human fibroblasts

Fibroblasts were isolated from skin biopsies obtained from selected patients at the Waisman Center at the University of Wisconsin-Madison per Institutional Review Board–approved human subject protocols. The following fibroblast lines were analyzed: FX08-01 (full mutation), FX08-02 (premutation), FX11-01 (full mutation), FX11-02 (premutation), FX13-01 (full mutation), FX13-02 (premutation), WC26 (double premutation), C0603 (control). In addition GM00497 (control) and GM00498 (control) fibroblasts from apparently unaffected individuals were purchased from the Coriell Cell Repository.

In the case of FX08-02, FX11-02, FX13-02 and C0603 cell lines the numbers of FMR1 CGG repeats were determined using a PCR-based protocol previously described [39]. The protocol combines gene-specific primers that flank the CGG repeat region of the FMR1 gene with gender-specific primers, a polymerase mixture, and a reaction buffer optimized for amplification of GC rich DNA. PCR was performed on an ABI Veriti thermal cycler (Applied Biosystems, Grand Island, NY). CGG repeat numbers were determined by agarose gel electrophoresis of PCR products and confirmed by capillary electrophoresis on an ABI 3730xl DNA Analyzer with POP-7 195 polymer using a 50-cm array [40], which is capable of defining exact CGG repeat number on samples with 200 CGG repeats or less.

In the case of WC26 and C0603 a different, previously described assay was used to determine the FMR1 CGG repeats [41]. Amplification of CGG repeats and flanking sequences was performed with AccuPrime Pfx DNA Polymerase (Thermo Fisher Scientific) in 15 microliter reactions containing 0.15 microliters of Pfx polymerase, 1x Reaction Mix, 2.5 M Betaine (Sigma), 7.5% DMSO (Sigma), 50 ng of genomic DNA, and 0.3 micromolar of each primer CGG-F (TCAGGCGCTCAGCTCCGTTTCGGTTTCA) and CGG-R (AAGCGCCATTGGAGCCCCGCACTTCC). Samples were amplified with an initial denaturation step of 98°C for 5 min; 35 cycles of 98°C for 35 s, 64°C for 35 s, and 68°C for 2 min, and a final extension step at 68°C for 5 min. PCR products were resolved on agarose gel electrophoresis. The CGG repeat number was estimated by comparing to DNA sizing ladder visualized on agarose gel electrophoresis.

### Western blotting

Expression of FMRP in fibroblast cell lines was analyzed by Western blotting with antibody to FMRP as described previously [42,43].

## Results

### CGG repeat and ribosome profiles for FMR1 RNA

**Fig 1:**
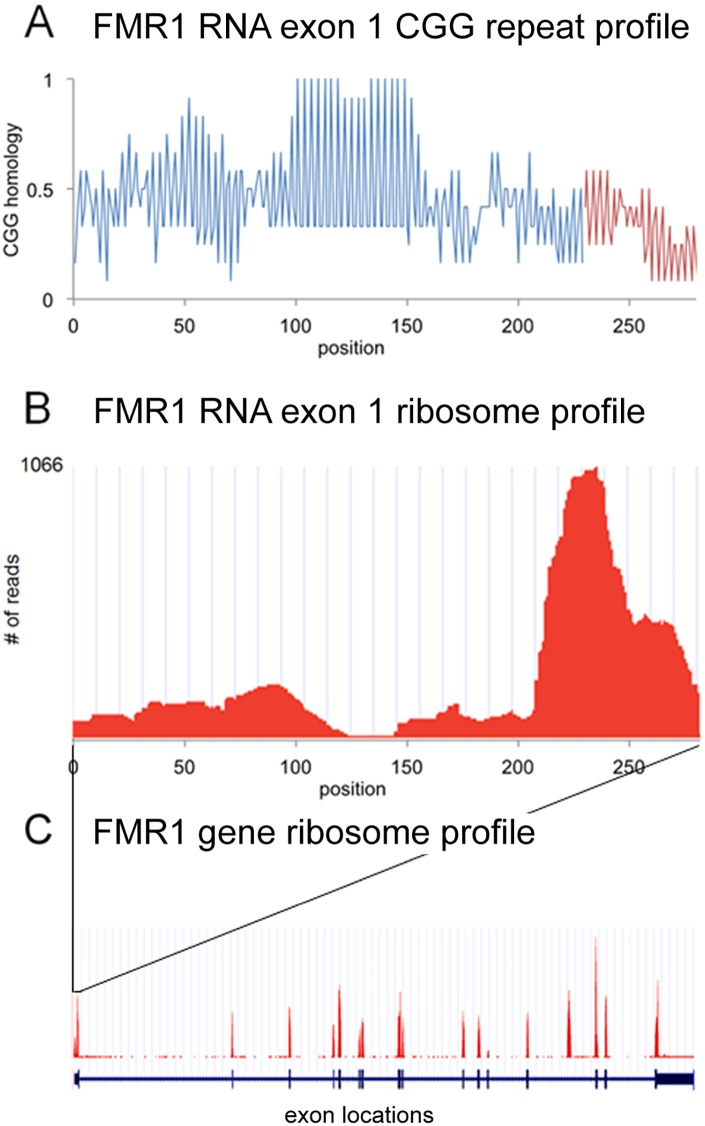
CGG repeat profile and ribosome profile for FMR1 RNA. Panel A shows the CGG profile for exon 1 of FMR1 RNA, including the 5’UTR (blue) and the initial portion the ORF (red), calculated as described in Materials and Methods. There is a region of CGG repeats between positions 100–150, interrupted by a single AGG at position 131. Panel B shows the corresponding ribosome profile for exon 1 of FMR1 RNA. The number of sequence reads at each position reflects the probability of ribosomes located at that position. Increased ribosome probability downstream of position 200 presumably reflects ribosomes engaged in conventional translation of the FMRP ORF beginning at the AUG at position 230. Increased ribosome probability in upstream regions (1–100 and 150–200) may reflect ribosomes initiating translation at non-canonical AUG-like sites upstream of the CGG repeats or ribosomes engaged in RAN translation of 5’UTR CGG repeats, consistent with reports that translation of CGG repeats in FMR1 RNA may require non-canonical AUG-like sequences upstream of the CGG repeats [11]. The paucity of reads in the region between positions 100–150, which corresponds to the CGG repeat region, may reflect inefficient sequencing through long regions of CGG repeats [24]. Panel C shows the ribosome profile for the entire FMR1 gene (red) and the locations of individual exons (blue), with 5’ and 3’UTR regions indicated by thinner lines and ORF regions indicated by thicker lines. The exon 1 region shown in panels A and B is indicated.

Fig 1 shows CGG repeat profiles and ribosome profiles for exon 1 of FMR1 RNA, which includes the 5’UTR and initial portion of the ORF. The CGG profile reveals a region of CGG repeats between positions 100–150 in the 5’UTR of FMR1 RNA. The ribosome profile reveals regions of increased ribosomal density between positions 200–250, presumably reflecting ribosomes engaged in conventional translation of the FMR1 ORF and between positions 0–125, which may reflect ribosomes stalled in the CGG repeat region in the 5’UTR of FMR1. The number of sequence reads in the CGG repeat region may underestimate the actual ribosome density in this region because sequencing through long sequences of CGG repeats is inefficient [24]. For comparison, the ribosome profile and exon locations for the entire FMR1 gene are shown in Fig 1 panel C. Ribosome densities for most exons are comparable, indicating that translational activity is relatively uniform across the ORF. Ribosome density in the 5’UTR is slightly lower compared to ribosome density in the ORF, indicating that RAN translation activity in the CGG repeat region is slightly lower than conventional translational activity in the ORF.

Expansion of CGG repeats in the 5’UTR of FMR1 RNA in premutation cells could potentially increase ribosome density in this region. This has not been tested directly because ribosome profiling has not been performed with premutation cells.

**Fig 2:**
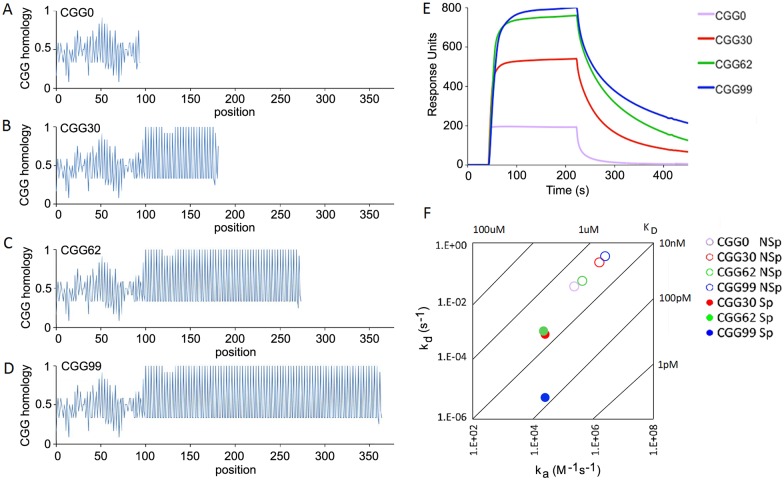
CGG repeat profiles and TMPyP4 binding for FMR1, CGG0, CGG30, CGG62 and CGG99 RNAs. Panels A-D—CGG repeat profiles for RNAs derived from the 5’UTR of FMR1 RNAs containing CGG 0, 30, 62, 99 were calculated using a sliding sequence algorithm (described in Materials Methods). Values of 1 in the profile indicate four consecutive CGG repeats in a row. Panel E—Binding of each CGG repeat RNA to serial dilutions of TMPyP4 was analyzed by SPR. Panel E shows representative SPR sensorgrams for binding of TMPyP4 to CGG 0, 30, 62 and 99 RNAs. Panel F—On-rates and off-rates determined by fitting SPR sensorgrams for each RNA to a heterogeneous ligand binding model. Apparent *K*_*D*_ values were calculated by dividing off rates by on rates NSp indicates non-specific binding, Sp indicates CGG-specific binding for each RNA.

TMPyP4, is a membrane-permeant tetraporphyrin ring compound that binds to, and disrupts, CGG repeat RNA secondary structure [13,14]. TMPyP4 binding to CGG repeat RNA was characterized by surface plasmon resonance (SPR) analysis with CGG repeat RNA as ligand and TMPyP4 as analyte, as shown in Fig 2. CGG repeat profiles for RNA transcripts derived from FMR1 RNAs with 0, 30, 62 and 99 CGG repeats are shown in panels A-D. In each transcript the 5’ flanking region from positions 1–101 in FMR1 RNA, corresponding to the region with increased ribosome density in Fig 1, is identical, followed by different numbers of CGG repeats (0, 30, 62, 99) for each transcript. To compare the TMPyP4 binding parameters for nonCGG repeat regions and for CGG repeat regions, SPR sensorgrams for TMPyP4 binding to RNAs with 0, 30, 62 and 99 repeats (shown in panel E) were globally fitted to a heterogeneous ligand binding model to determine non-specific and specific binding parameters for each transcript (shown in panel F). One set of binding parameters is similar for all transcripts (*K*_*D*_ ~ 0.5 micromolar), presumably corresponding to non-specific binding of TMPyP4 to common nonCGG repeat regions present in each transcript. In addition, CGG 30, 62 and 99 RNAs, exhibit a second set of binding parameters, presumably corresponding to specific binding of TMPyP4 to different number of CGG repeats in each transcript. Apparent on-rates for CGG-specific binding are similar for CGG 30, 62 and 99 RNAs (*k*_*a*_ ~ 2 x 10^4^ M^-1^s^-1^) but apparent off-rates are slower for CGG99 RNA (*k*_*d*_ ~ 2 x 10^−6^ s^-1^) compared to CGG30 and CGG62 RNAs (*k*_*d*_ ~ 2 x 10−^4^ s^-1^). This may reflect tighter binding of TMPyP4 to CGG 99 RNA compared to CGG 30 and CGG 62 RNAs, possibly because of increased numbers of CGG repeats, or alternatively, may reflect rebinding of TMPyP4 to CGG99 RNA during the dissociation phase. In any case, these results indicate that TMPyP4 binds non-specifically to nonCGG repeat sequences and specifically to CGG repeat sequences. Overall the apparent *K*_*D*_ for TMPyP4 binding to all RNAs is < 1 micromolar. Accordingly, in subsequent experiments, cells were incubated with TMPyP4 at a final concentration of 1 micromolar to ensure saturation binding to all CGG repeat RNA molecules in the cell.

### CGG repeat RNA molecules and Venus-ARC RNA molecules are co-localized in granules and CGG repeat RNA inhibits translation of Venus-ARC RNA in the same granule

**Fig 3:**
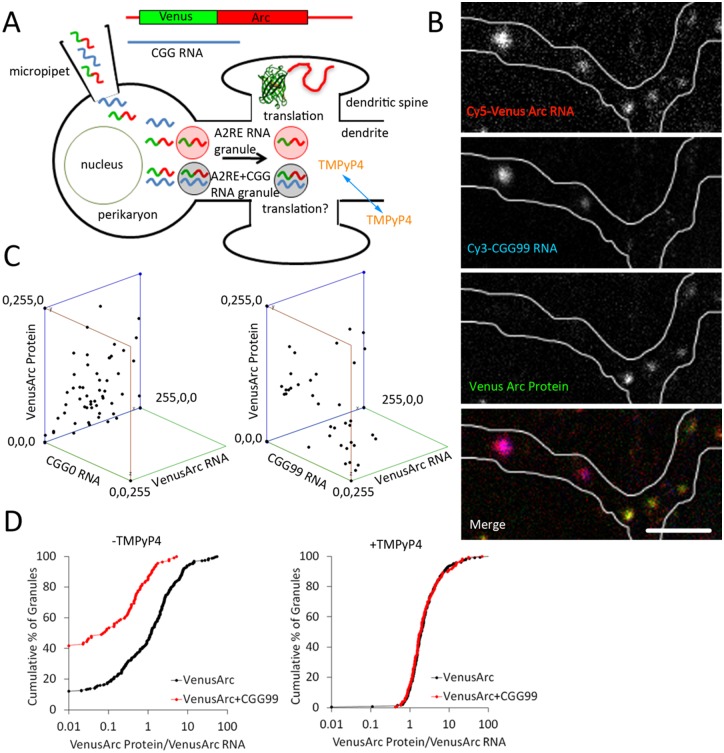
CGG repeat RNA and ARC RNA are co-localized in granules and CGG repeat RNA inhibits translation of ARC RNA in hippocampal neurons. Panel A shows a schematic outline of the experimental protocol. Differentially labeled CGG repeat RNA and Venus-ARC RNA were co-injected into the cytoplasm of hippocampal neurons where they are localized in granules in dendrites. Venus-ARC RNA is translated in granules near dendritic spines. In some cases hippocampal neurons were incubated with membrane-permeant TMPyP4 before injecting RNA. Panel B shows the distribution of Venus-ARC RNA (red), CGG99 RNA (blue) and newly-synthesized Venus-ARC protein (green) in discrete granules in a three channel confocal images of a dendritic segment. Seven granules are visible in this dendritic segment, all of which contain Venus-ARC RNA, two of which also contain CGG99 RNA, and five of which lack detectable CGG99 RNA. Newly synthesized Venus-ARC protein is detected in the five granules lacking CGG99 RNA but not in the two granules containing CGG99 RNA. Scale bar indicates 1 micrometer. Panel C shows 3-dimensional graphs of fluorescence intensities for Venus-ARC RNA, Venus-ARC protein and CGG99 (right panel) or CGG0 (left panel) RNAs in a population of individual granules in hippocampal neurons. Panel D shows Kolmogorov-Smirnov cumulative frequency plots of specific translational activities (Venus-ARC protein/Venus-ARC RNA) for granules containing detectable (defined as integrated fluorescence intensity > 10 arbitrary units) CGG99 RNA (red), or in granules where CGG99 RNA was undetectable (defined as integrated fluorescence intensity < 10 arbitrary units) (black),. Specific translational activities were measured for granules in untreated (left panel) or in TMPyP4-treated (right panel) hippocampal neurons.

FMR1 RNA containing CGG repeats is localized and translated in RNA granules [19]. To determine if CGG repeats by themselves are sufficient for RNA localization in granules and if CGG repeat RNA affects translation of other RNAs localized in the same granules, CGG0 and CGG99 RNAs were microinjected into hippocampal neurons, along with Venus-ARC RNA, as a marker for granules and as a reporter for granule translation. CGG0 and CGG99 RNAs were labeled by in vitro transcription with Cy3-UTP and Venus-ARC RNA was differentially labeled by in vitro transcription with Cy5-UTP. Co-injected neurons were incubated for 2 hours to allow time for localization and translation of the injected RNAs in individual granules. Fluorescent Venus-ARC RNA, fluorescent CGG repeat RNA and fluorescent newly synthesized Venus-ARC protein were imaged simultaneously by three channel confocal microscopy. The relative amounts of each component associated with each granule were quantified by integrating fluorescent intensities in each channel for each granule. In some experiments neurons were incubated with TMPyP4, prior to microinjection, to disrupt secondary structure in CGG repeat RNA. The experimental protocol is outlined schematically in Fig 3A.

The experimental protocol outlined Fig 3A is based on several considerations. Hippocampal neurons were used to analyze localization and translation in granules because individual granules are well resolved in neuronal dendrites. Venus-ARC RNA was used as a marker for granules because it contains an A2RE sequence that binds to hnRNP A2 and mediates localization in granules [18]. It also serves as a reporter for translation in granules because Venus-ARC RNA is translated in granules and newly-synthesized Venus-ARC protein molecules remain in the vicinity of the granules where they are synthesized [19]. Truncated CGG repeat RNAs derived from the 5’UTR of FMR1 RNA but lacking the FMRP ORF, were injected to test the effect of CGG repeat RNA itself, without potential confounding effects of FMRP protein. CGG0 and CGG99 RNAs were used because they do not represent substrates for conventional translation, since they lack initiation and termination codons, although the region upstream of the CGG repeats in FMR1 does contain several non-canonical AUG-like sequences that could potentially mediate translation initiation at low efficiency [11]. Because CGG99 contains CGG repeats it is localized in granules and may represent a substrate for RAN translation. CGG repeats can also form secondary structures, which may cause ribosomes to stall in the vicinity of the CGG repeats. Fluorescent RNAs were injected directly into the cytoplasm rather than into the nucleus in order to assay effects of CGG repeat RNA on cytoplasmic translation, while avoiding effects of CGG repeats on nuclear processes, such as nuclear inclusion formation [44], nuclear processing or nuclear transport, that could potentially affect subsequent translation or decay in cytoplasm. There are several reasons to believe that CGG repeat RNA is not degraded during the course of the experiment. Eucaryotic cells have multiple different decay pathways for degrading dysfunctional RNAs including: nonsense mediated decay (NMD), triggered by premature nonsense codons in the RNA [45], non-stop decay (NSD), triggered by lack of a termination codon preceding the polyA tail on the RNA [46], and no-go decay (NGD), triggered by stalled elongation on the RNA [47]. CGG repeat RNAs do not represent substrates for NMD, because they lack termination codons or for NSD, because they lack a polyA tail. CGG repeat RNAs could potentially represent substrates for NGD, if translation elongation is stalled by CGG repeat secondary structure in the injected RNA. However injection of fluorescent RNA into the cytoplasm provides an internal control for RNA degradation because fluorescent fragments produced by RNA decay in the cytoplasm are translocated to the nucleus [18]. In this experiment none of the injected cells showed detectable nuclear fluorescence, indicating that RNA injected into the cytoplasm was not degraded during the time course of the experiment. RNA localization and translation in granules were measured 2 hours after microinjection in order to analyze primary effects of CGG repeat RNA on granule translation, while minimizing potential secondary effects due to RNA toxicity, decay or changes in cellular physiology caused by long term presence of exogenous CGG repeat RNA in the cell.

Fig 3 panel B shows several individual granules in a representative dendritic segment from a microinjected neuron and Fig 3 panel C shows total integrated fluorescence intensities for microinjected Venus-ARC RNA, CGG99 RNA (right panel) or CGG0 RNA (left panel) and newly-synthesized Venus-ARC protein in multiple individual granules. Venus-ARC RNA is detected in all RNA granules, CGG99 RNA is detected in a subset of Venus-ARC RNA granules and Venus-ARC protein is detected in granules that do not contain detectable CGG99 RNA, but not in granules that contain detectable CGG99 RNA, indicating that CGG repeat RNA inhibits translation of Venus-ARC RNA localized in the same granule. Microinjected CGG0 RNA is not detected in granules and does not affect synthesis of Venus-ARC protein in granules. These results indicate that CGG repeat RNA is localized in granules and inhibits translation of Venus-ARC RNA in the same granules.

To quantify specific translational activities in individual granules, Venus-ARC RNA and newly-synthesized Venus ARC protein were measured in multiple granules, with and without detectable CGG99 RNA (Fig 3, panels C and D). Specific translational activities were calculated by dividing the amount of newly-synthesized Venus-ARC protein by the amount of Venus-ARC RNA in each granule. Individual granules exhibit variable translational activities, which is consistent with previous single molecule imaging observations of translational output from individual granules [19]. In order to compare translational activities for a large population of granules the results are presented in the form of a Kolmogorov-Smirnov plot showing cumulative frequencies for granules with different specific translational activities. Granules without detectable CGG99 RNA (black symbols) exhibit a range of translational activities (0.01–50), with a small proportion (~10%) of translationally-inactive granules. Variation in translational activity among granules without exogenous CGG99 RNA may be due to variable amounts of endogenous CGG repeat RNAs in different granules. Granules with detectable CGG99 RNA (red symbols) exhibit reduced translational activities (0.01–5) with a larger proportion (~40%) of translationally-inactive granules. This indicates that CGG99 RNA localized in granules inhibits translational activity of Venus-ARC RNA localized in the same granules.

TMPyP4, binds to CGG repeat RNA and disrupts CGG repeat secondary structure. To determine if TMPyP4 rescues granule translation, neurons were treated with TMPyP4 prior to injection with Cy5-labeled Venus-ARC RNA, by itself or with CGG99 RNA (Fig 3 panel D). In TMPyP4–treated cells, the ranges of translational activities in individual granules, with detectable CGG99 RNA (red symbols) or without detectable CGG99 RNA (black symbols), are similar (the cumulative frequency plots are superimposable) and translationally-inactive granules are not detected in either case. Since TMPyP4 binds specifically to CGG repeats and since Venus-ARC RNA does not contain CGG repeats, and since TMPyP4 differentially affects translation of granules containing CGG repeat RNA, the effect of TMPyP4 on translation of Venus-ARC RNA may be mediated by its binding to CGG repeat RNA molecules in the same granules. These results indicate that TMPyP4 rescues translation of Venus-ARC RNA in granules containing CGG99 RNA.

**Fig 4:**
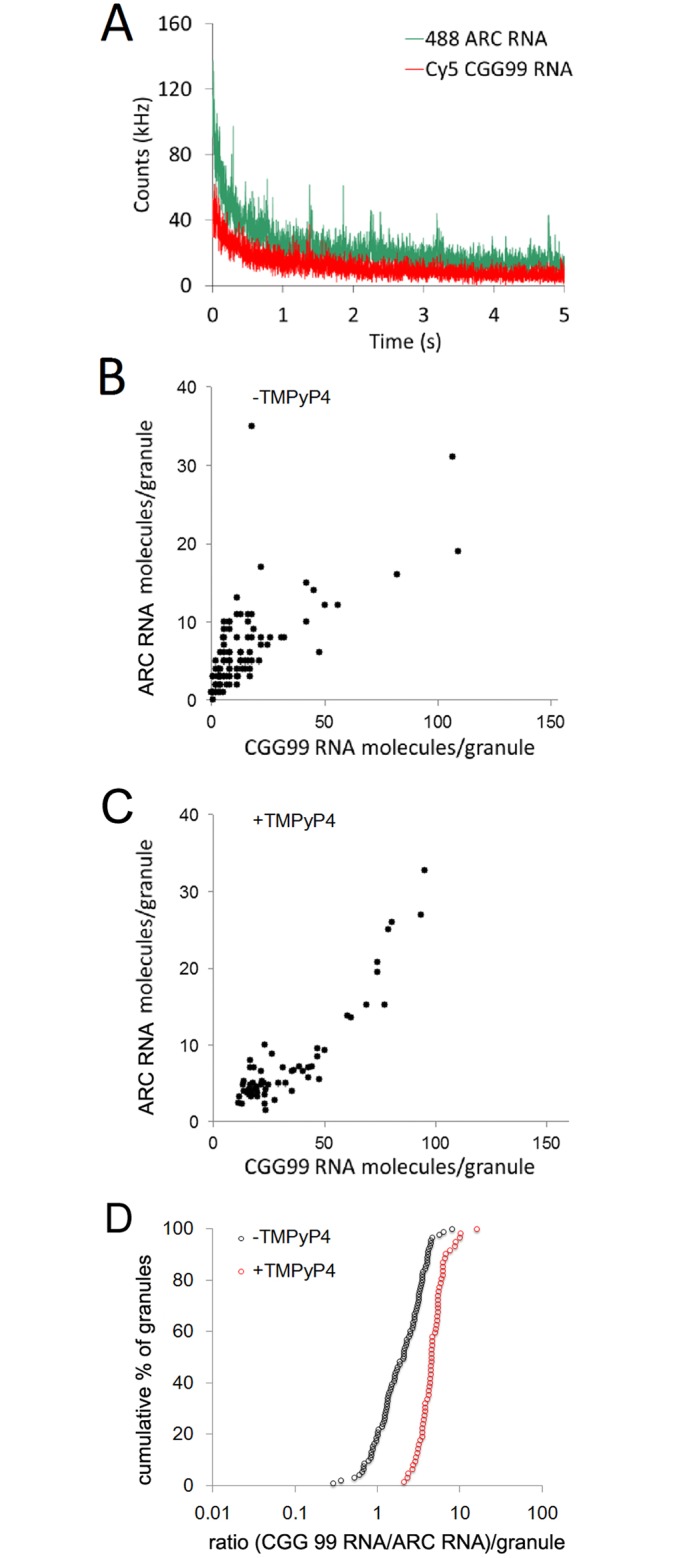
FCS photobleaching of ARC RNA and CGG 99 RNA molecules in individual granules in hippocampal neurons. Panel A—the FCS observation volume was positioned to encompass a single individual immobile granule containing differentially labeled fluorescent ARC RNA and CGG99 RNA molecules in a hippocampal neuron. Continuous illumination during the FCS measurement results in photobleaching of fluorescent RNA molecules of each type in the granule, which is recorded as count rate decay in each FCS channel. The numbers of fluorescent RNA molecules of each type in the granule are determined by dividing the total decay in counts during photobleaching by the counts per molecule for each RNA determined by FCS in solution. Panel B shows a scatter plot for numbers of ARC RNA and CGG99 RNA molecules in individual granules in hippocampal neurons in the absence of TMPyP4. Panel C shows a scatter plot for numbers of ARC RNA and CGG99 RNA molecules in individual granules in hippocampal neurons in the presence of TMPyP4. Panel D shows Kolmogorov-Smirnov plots of the ratios of CGG99 RNA molecules to ARC RNA molecules in individual granules in the absence (black symbols) and presence (red symbols) of TMPyP4.

If CGG repeat RNA inhibits translation of other RNAs in the same granule, the magnitude of inhibition may reflect the number of CGG repeat RNA molecules relative to other RNA molecules in the same granule. The numbers of CGG repeat RNA and ARC RNA molecules in individual granules were determined by fluorescence correlation spectroscopy (FCS) photobleaching (Fig 4) [37,38]. Alexafluor 488-labeled ARC RNA and Cy5-labeled CGG99 RNA were first analyzed by FCS in solution to determine fluorescence counts per molecule for each RNA. For these experiments ARC RNA was used instead of Venus-ARC RNA in order to avoid fluorescence from newly-synthesized Venus-ARC protein. The numbers of Alexafluor 488-ARC RNA and Cy5-CGG99 RNA molecules in individual granules in live cells were determined by positioning the FCS volume to encompass a single immobile RNA granule and recording decay of counts over time in each channel due to photobleaching of fluorescent RNA molecules in the immobile granule during the FCS measurement. Dividing the total loss of counts per granule by counts per individual RNA molecule provides a measure of the number of RNA molecules of each type in the granule encompassed by the FCS volume (Fig 4 panel A). Endogenous CGG repeat or A2RE RNA molecules in individual granules are not detected by FCS photobleaching because they are not fluorescent.

In the absence of TMPyP4 (Fig 4 panel B), the relative numbers of ARC RNA molecules and CGG99 RNA molecules per granule are correlated over the population of granules but the absolute number of CG99 RNA molecules is greater than the number of ARC RNA molecules in most granules. This suggests that CGG99 RNA molecules and ARC RNA molecules are assembled into granules by similar mechanisms but CGG 99 RNA is assembled more efficiently than ARC RNA. In the presence of TMPyp4 (Fig 4 panel C) the number of CGG99 RNA molecules compared to ARC RNA molecules is slightly increased. Fig 4 pane; D shows the ratios of CGG99 RNA molecules to ARC RNA molecules for granules in the absence and presence of TMPyP4. In the absence of TMPyP4 the ratio varies from 1–10, indicating that CGG99 RNA molecules are assembled into granules more efficiently than ARC RNA molecules. CGG99 RNA contains multiple CGG repeat sequences that can potentially bind to multiple hnRNP A2 molecules, which may mediate more efficient assembly into granules, while ARC RNA contains a single A2RE sequence that binds to a single hnRNP A2 molecule, which may mediate less efficient assembly into granules. In the presence of TMPyP4 the ratios are shifted slightly to higher values indicating that assembly of CGG99 RNA molecules into granules is slightly enhanced. Disruption of secondary structure by TMPyP4 may increase binding of hnRNP A2 molecules to CGG repeat RNA molecules, which could facilitate more efficient assembly into RNA granules. This could result in increased hnRNP A2 concentration in CGG99 RNA granules in the presence of TMPyP4 but this was not tested experimentally.

In the absence of TMPyP4 the range of values for the ratio of CGG99 RNA to ARC RNA in individual granules (1–10) is narrower than the range of values for translational activities in CGG99 containing granules (0.01–5) (compare Figs 4B to 3D). This indicates that inhibition of granule translation by CGG repeat RNA is not linearly proportional to the ratio of CGG99 RNA and ARC RNA in individual granules. This non-linearity could be due to CGG repeat RNA affecting translation of other endogenous RNAs that encode proteins known to regulate translation [1]. If CGG99 RNA inhibits translation of such endogenous RNAs this could amplify the effect of CGG99 RNA on translation of ARC RNA in granules.

### CGG repeat expansions in endogenous FMR1 RNA inhibit translation of Venus-ARC RNA in premutation human fibroblasts

**Fig 5:**
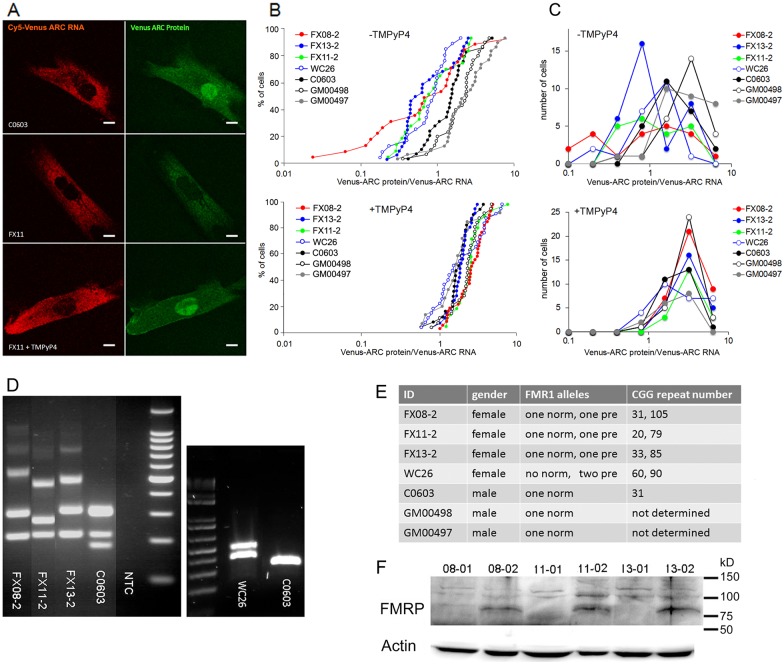
CGG repeat expansions in the FMR1 gene inhibit translation of Venus-ARC RNA in human fibroblasts. Panel A—Human fibroblasts from control individuals C0603 (control male, 31 repeats), GM00497 (control male, unknown repeat number), GM00498 (control male, unknown repeat number) and FMR1 premutation carriers FX08-2 (female, 31, 105 repeats), FX11-2 (female, 20, 79 repeats), FX13-2 (female, 33, 85 repeats), and WC26 (female, two premutation alleles, 60, 90 repeats) were microinjected with Venus-ARC RNA and after 2 hours Venus-ARC RNA and newly-synthesized Venus-ARC protein were imaged by dual channel confocal microscopy. Representative images are shown for untreated control and premutation cells and for premutation cells treated with TMPyP4. Scale bars indicate 5 micrometers. Panel B—Cumulative Kolmogorov Smirnov plots for specific translational activities (newly-synthesized Venus-ARC protein/microinjected Venus-ARC RNA) in individual cells for 3 control and 4 premutation fibroblast cell lines as described for Panel A, untreated (top) and treated with TMPyP4 (bottom). Panel C—Frequency distribution plots for specific translational activities (newly-synthesized Venus-ARC protein/microinjected Venus-ARC RNA) in cells for 3 control and 4 premutation fibroblast cell lines as described for Panel B, untreated (top) and treated with TMPyP4 (bottom). Panel D—PCR analysis of CGG repeat numbers in FX08-02, FX11-02, FX13-02 and C0603 fibroblasts with a 100 bp DNA ladder (left panel) and in WC26 and C0603 fibroblasts with a 1 kb ladder (right panel). In the panel on the left, in the FX08-2, FX11-2, and FX13-2 lanes, the band at the bottom of the gel represents female gender specific PCR product, the bands immediately above the gender specific band represent PCR products from CGG repeat alleles in the normal range and the bands above represent PCR products from expanded CGG repeat alleles. The fainter products near the top of the gel are of unknown origin. In the C0603 lane, the two bands near the bottom of the gel represent male and female gender specific PCR products, and the band above the gender specific bands represents PCR product from the normal CGG repeat allele. In the panel on right, the two bands in the WC26 lane both represent PCR products from expanded CGG repeat alleles and the single band in the C0603 lane represents PCR product from the CGG repeat allele in the normal range. Panel E—Table showing ID, gender, FMR1 alleles and CGG repeat numbers (based on panel D) for each cell line. Panel F—Western blotting of FMRP expression in full mutation (FX08-1, FX11-01, FX13-01) and premutation (FX08-2, FX11-02 and FX13-02) cell lines with actin loading controls.

The effect of CGG repeat expansions in endogenous FMR1 RNA on translation of Venus-ARC RNA was analyzed by microinjecting Venus-ARC RNA labeled with Cy5-UTP into human fibroblasts from premutation carrier individuals with different numbers of CGG repeats in the endogenous FMR1 gene (Fig 5). Microinjected Venus-ARC RNA and newly-synthesized Venus-ARC protein were imaged in multiple cells by dual channel confocal microscopy two hours after injection to allow time for injected RNA to be translated. Fluorescent Venus-ARC RNA was restricted to the cytoplasm and appeared to have a granular distribution, suggesting that the injected RNA was localized in granules and was not degraded (Fig 5 panel A). However in most cells discrete individual granules were not sufficiently well resolved from each other to quantify RNA in individual granules. Newly-synthesized Venus-ARC protein accumulates in both cytoplasm and nucleus of fibroblasts, as reported previously [48].

Total fluorescent RNA and total fluorescent protein were integrated over the entire cell as a measure of the total amount of injected Venus-RNA and the total amount of newly-synthesized Venus-ARC protein, respectively, in each cell. Specific translational activities were calculated by dividing the amount of Venus-ARC protein by the amount of Venus-ARC RNA in each cell. The results for multiple individual cells from 3 control and 4 premutation fibroblast cell lines are shown in cumulative Kolmogorov-Smirnov plots (Fig 5 panel B). In control fibroblasts (C0603, GM00497, GM00498) from individuals with normal numbers of CGG repeats in the FMR1 gene, most cells exhibit translational activities between 1–10. Intrinsic variation in control cells may be due to cell-to-cell variations in expression of other endogenous CGG repeat RNAs that affect translation in these cells. In premutation fibroblasts (FX08-2, FX11-2, FX13-2) from individuals with CGG repeat expansions in one copy of the FMR1 gene, some cells exhibit translational activities comparable to control cells (1–10) while other cells exhibit reduced translational activities (0.02–1). The translational activities in the second sub-population exhibit the same rank order as the number of CGG repeats in the expanded FMR1 allele in the different cell lines (FX08>FX13>FX11), suggesting that inhibition of translational activity is proportional to CGG repeat number in different cell lines. In cells from one individual with CGG repeat expansions in both FMR1 alleles (WC26), specific translational activities are reduced in all cells.

To determine if TMPyP4 rescues translation in premutation fibroblasts, cells were treated with TMPyP4 for 24 hours prior to injection with Venus-ARC RNA. After TMPyP4 treatment, control and premutation fibroblasts exhibit comparable specific translational activities, which are similar to untreated control cells. This indicates that TMPyP4 rescues translation in premutation fibroblasts from individuals with CGG repeat expansions in the endogenous FMR1 gene, which is consistent with the effect of TMPyP4 on granule translation in neurons microinjected with exogenous CGG repeat RNA. These results indicate that translation of Venus-ARC in human fibroblasts is inhibited by CGG repeat expansions in FMR1 RNA and rescued by TMPyP4.

The existence of two subpopulations of cells with different translational activities in premutation carriers could reflect random X inactivation of different FMR1 alleles in different sub-populations of fibroblasts. In cells with reduced translational activities, the FMR1 allele with expanded CGG repeats may be active, while in cells with normal translational activities, the FMR1 allele with normal CGG repeat numbers may be active. In WC26 cells, with reduced translational activities in all cells, either FMR1 allele may be active because both alleles contain CGG repeat expansions. To test this possibility, translational activities in different cell lines were plotted as a frequency distribution plot (Fig 5 panel C). Control cells (C0603, GM00497, GM00498) with a single FMR1 allele with normal numbers of CGG repeats, exhibit a unimodal distribution with normal translational activities. Premutation cells (FX08-2, FX11-2, FX13-2) with one normal and one FMR1 allele with expanded CGG repeats exhibit a bimodal distribution of translational activities (one normal and one reduced) and the double premutation cells (WC26) with expanded CGG repeats in both FMR1 alleles, exhibit a unimodal distribution with reduced translational activities in all cells. In cells treated with TMPyP4 all cell lines exhibit unimodal distributions with normal translational activities. These results indicate that in premutation carrier cells with one normal FMR1 allele and one premutation FMR1 allele with expanded CGG repeats, translational activity is determined by the active FMR1 allele in each cell.

FMRP is a translational repressor that inhibits ARC translation. Levels of FMRP expression are generally not affected much in premutation cells [42,43]. However, variation in Venus-ARC translation in different premutation fibroblast cell lines could conceivably reflect variation in endogenous FMRP expression in these specific lines. Western blotting showed comparable levels of FMRP expression in FX08-2, FX11-2 and FX13-2 cell lines (Fig 5 panel F). This analysis would not necessarily detect variations of FMRP expression due to X-inactivation of different FMR1 alleles in different subpopulations of cells. Since expanded CGG repeats in FMR1 RNA may reduce expression of FMRP from the downstream ORF, subpopulations of cells where the FMR1 allele with expanded CGG repeats is active might have reduced FMRP expression, which would be expected to result in increased ARC translation. However the results show decreased ARC translation in these cells. Moreover, TMPyP4 increases FMRP expression from FMR1 RNA with expanded CGG repeats, which might decrease ARC translation. However the results show increased ARC translation in the presence of TMPyP4. For these reasons it is unlikely that variation in Venus-ARC translation in different cell lines is primarily due to variations in FMRP expression.

### CGG repeat expansions in the FMR1 gene cause increased calcium transients in human fibroblasts

**Fig 6:**
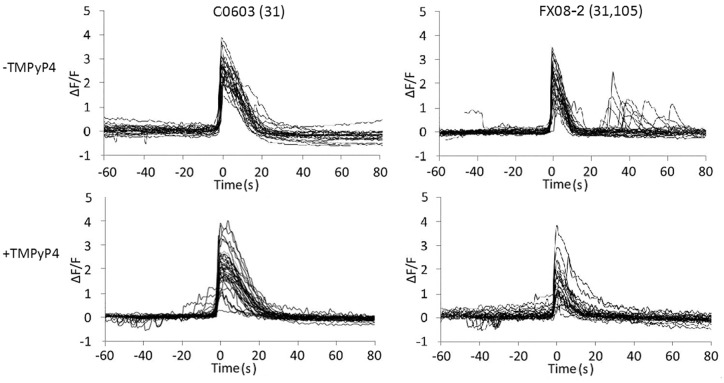
CGG repeat expansions in the FMR1 gene disrupt regulation of calcium transients in human fibroblasts. Control (C0603) and premutation (FX08, 31,105 repeats) fibroblasts were loaded with fluorescent calcium indicator Fluo-4 and incubated with bradykinin to induce calcium transients. Cells were imaged by time-lapse confocal microscopy to visualize changes in intracellular calcium concentrations over time. Total fluorescence intensity over the entire cell was integrated in each time frame for 20 control and 20 premutation cells, in the absence or presence of TMPyP4. F/F_0_ values for each cell are plotted, with initiation of primary calcium transients aligned at time = 0 s.

Expanded CGG repeats in endogenous FMR1 RNA inhibit translation of exogenous Venus-ARC RNA, which is a reporter for translation of RNAs localized in the same granules as FMR1 RNA. Translation of endogenous RNAs localized in the same granules may also be inhibited, which may result in additional cellular phenotypes. In this regard, previous studies have shown that calcium transients are increased in astrocytes from CGG KI mice, which contain a CGG repeat transgene [21], and in iPSC-derived neurons from individuals with expanded CGG repeats in the endogenous FMR1 gene [22]. These observations suggest that expanded CGG repeat RNA inhibits translation of endogenous RNAs encoding proteins that mediate calcium homeostasis. To determine if calcium transients are affected in fibroblasts from individuals with CGG repeat expansions in the endogenous FMR1 gene, control and premutation human fibroblasts were loaded with Fluo-4 as a calcium indicator and bradykinin was added to induce calcium transients (Fig 6). Fluo-4 fluorescence in individual cells was imaged by time-lapse confocal microscopy. Calcium transients were detected by integrating Fluo-4 fluorescence over each cell at each time point. In control fibroblasts (C0603), bradykinin induced a single primary calcium transient, which returned to baseline after ~ 20 sec in all cells. In premutation fibroblasts with expanded CGG repeats in the endogenous FMR1 gene (FX08-2), the primary calcium transient was followed by secondary calcium transients in some cells. The time interval between primary and secondary calcium transients was variable (20–60 sec) among different cells. These results indicate that CGG repeat expansions in the endogenous FMR1 gene disrupt calcium homeostasis in premutation fibroblasts. Premutation cells that did not exhibit secondary calcium transients may represent cells in which the normal FMR1 allele is active and the allele with expanded CGG repeats is inactivated.

To determine if TMPyP4 rescues calcium homeostasis in premutation fibroblasts, cells were treated with TMPyP4 for 24 hours before analyzing brakykinin-induced calcium transients. In TMPyP4-treated control and premutation fibroblasts, bradykinin induced a single primary calcium transient in all cells with no secondary transients. This indicates that TMPyP4 rescues calcium homeostasis in premutation fibroblasts and that CGG repeat expansions in the endogenous FMR1 gene are associated with a phenotype that is manifested in fibroblasts.

The control fibroblasts used in these experiments were obtained from apparently normal male individuals with normal numbers of CGG repeats in the FMR1 gene, while the premutation fibroblasts were obtained from apparently normal female individuals carrying premutation CGG expansions in the FMR1 gene. This raises the possibility that the observed differences in granule translation and calcium homeostasis between control and premutation fibroblasts could reflect gender differences rather than differences in CGG repeat expansions in the FMR1 gene. We believe this possibility is unlikely for several reasons. First, in cells with one normal FMR1 allele and one premutation allele, some cells exhibit normal granule translation and calcium homeostasis, while others exhibit reduced granule translation and disrupted calcium homeostasis, while in cells where both FMR1 alleles contain CGG repeat expansions, all cells exhibit reduced granule translation. Since the FMR1 gene is located on the X chromosome, which is subject to random X inactivation in different cells, this suggests that granule translation and calcium homeostasis are regulated by the number of CGG repeats in the active FMR1 allele in each cell, rather than by the gender of the donor. Second, the extent of inhibition of granule translation in premutation cells is proportional to the number of CGG repeats in the premutation allele of the FMR1 gene, suggesting that granule translation is regulated by the number of CGG repeats in the FMR1 gene rather than by gender differences. Third, both translation and calcium homeostasis in premutation cells are rescued by TMPyP4, which binds to CGG repeat RNA and destabilizes CGG repeat secondary structure, suggesting that both translation and calcium homeostasis are regulated by CGG repeats rather than by gender differences. For these reasons we believe the differences in granule translation and calcium transients between control and premutation human fibroblasts observed in this study are likely due to differences in CGG repeat number in the endogenous FMR1 gene rather than to gender differences.

## Discussion

CGG repeat RNAs, such as FMR1 RNA, and A2RE RNAs, such as ARC RNA, are localized and translated in the same RNA granules. CGG repeat RNA inhibits translation of ARC RNA in the same granule and TMPyP4 rescues ARC RNA translation in granules containing CGG repeat RNA. These observations can be explained by multiple potential mechanisms involving component(s) in granules that: 1) regulate translation; 2) are affected by CGG repeats; and 3) whose effects are reversed by TMPyP4. Here we will discuss three potential mechanisms involving components that meet these criteria: hnRNP A2, ribosomes, and EIF2AK2,.

The first potential mechanism involves hnRNP A2, which binds to CGG repeat RNA [15–17] and is known to enhance translation of A2RE RNAs in granules [49]. If RNA molecules with expanded CGG repeats bind increased numbers of hnRNP A2 molecules this could potentially reduce the number of hnRNP A2 molecules available to bind to ARC RNA molecules, which could potentially reduce translation of ARC RNA in granules. Several considerations argue against this possibility. First, the concentration of hnRNP A2 in cytoplasm is quite high (6 micromolar), which suggests that this component may not be rate limiting for translation of ARC RNA. Second, since hnRNP A2 is required for assembly of ARC RNA into granules, reduced binding of hnRNP A2 to ARC RNA might result in reduced assembly of ARC RNA into granules, which was not observed (Fig 4). Third, TMPyP4 rescues translation but is not known to interact with hnRNP A2. For these reasons it seems unlikely that intragranule competition between CGG repeat RNA and A2RE RNA for hnRNP A2 is responsible for inhibition of ARC RNA translation by CGG repeat RNA in the same granule.

The second potential mechanism involves ribosomes, which are required for translation of ARC RNA and also accumulate in the vicinity of CGG repeats. Ribosome profiling reveals increased ribosome density in the vicinity of CGG repeats in the 5’UTR of FMR1. If the number of CGG repeats is expanded, the ribosome density in this region could be further increased, although this has not been tested experimentally. Increased ribosome density in the CGG repeat region might reduce the number of ribosomes available for translation of ARC RNA in the same granule. The magnitude of such an effect would depend on the relative number of ribosomes associated with expanded CGG repeats in the 5’UTR of premutation FMR1 RNA molecules compared to the total number of ribosomes in each granule. In this regard, single molecule imaging reveals that granules with increased numbers of RNA molecules do not have increased numbers of translational events per granule [19], which implies that the number of ribosomes per granule may be rate limiting for translation. Furthermore, if secondary structure in CGG repeat RNA causes ribosomes to accumulate in the 5’UTR of FMR1 RNA, TMPyP4 might reduce ribosome density by disrupting CGG repeat secondary structure in this region, which might increase availability of ribosomes for translation of ARC RNA in the same granules, reversing the effects of CGG repeat expansion. These considerations are consistent with intragranule competition between CGG repeat RNA and ARC RNA for ribosomes as a potential mechanism for inhibition of ARC RNA translation by CGG repeat RNA in the same granule.

The third potential mechanism involves EIF2AK2, eukaryotic translation initiation factor 2 alpha kinase 2, a serine/threonine protein kinase activated by dsRNA that inhibits conventional translation by phosphorylating translation initiation factor EIF2alpha [50]. EIF2AK2 RNA itself contains CGG repeats in the 5’UTR [1], which may mediate localization and translation of EIF2AK2 RNA in the same granules as FMR1 RNA and ARC RNA. CGG repeats in FMR1 RNA can form dsRNA secondary structure, which may activate EIF2AK2, thereby inhibiting ARC RNA translation in the same granule. Expansion of CGG repeats in FMR1 RNA may increase stability of dsRNA secondary structure, thereby increasing activation of EIF2AK2 and reducing translation of ARC RNA. TMPyP4 may destabilize CGG repeat dsRNA secondary structure in FMR1 RNA, thereby reversing activation of EIF2AK2 and rescuing translation of ARC RNA. These considerations are consistent with activation of EIF2AK2 by CGG repeat RNA as a potential mechanism for inhibition of ARC RNA translation in the same granule.

The above, and other, potential mechanisms could be distinguished by knocking out or inhibiting specific components that are: localized in the same granules as FMR1 RNA, regulate translation of other RNAs in the same granule, are potentially affected by CGG repeat expansions in FMR1 RNA, and whose effects are potentially reversed by TMPyP4. However, without such experimental validation it is not possible to identify specific molecular mechanism(s) for the observations reported here.

The human exome contains multiple different CGG repeat RNAs besides FMR1 RNA and multiple different A2RE RNAs besides ARC RNA, some of which may be localized in the same RNA granules as FMR1 RNA because CGG repeat sequences and A2RE sequences both bind to hnRNP A2, which mediates granule assembly. This may create an intra-granule translational regulatory network, where expansion of CGG repeats in one RNA increases the overall CGG repeat burden in the granule, thereby decreasing translation of other RNAs in the same granule. Since CGG repeat RNAs and A2RE RNAs encode multiple different proteins that mediate multiple different cellular functions, regulating translation of these RNAs in granules could have pleiotropic effects on the cell. In this regard, expression of a CGG repeat transgene is known to affect translation of multiple different RNAs in mice and flies [20], which could explain why expansions of CGG repeats in various genes are associated with pleiotropic pathological manifestations such as: late onset, tremors, ataxia, dysphagia (difficulty swallowing), dysarthria (difficulty speaking).

Calcium homeostasis is a complex cellular process mediated by multiple different proteins, some of which are encoded by CGG repeat and/or A2RE RNAs. For example: CGG repeat RNAs such as: CACNA1A (voltage dependent calcium channel) and KCNMA1 (calcium activated potassium channel), could potentially affect calcium homeostasis and A2RE RNAs such as CAMK2A (calcium calmodulin-dependent protein kinase) and NRGN (neurogranin/calmodulin binding protein) could also potentially affect calcium homeostasis. If calcium homeostasis is mediated by proteins encoded by CGG repeat and/or A2RE RNAs that are localized in granules and translationally regulated by CGG repeat RNA this could explain why calcium homeostasis is disrupted in premutation fibroblasts from individuals with CGG repeat expansions in the endogenous FMR1 gene. However it is difficult to identify one specific gene or group of genes that mediates the calcium phenotype in premutation fibroblasts because multiple different CGG repeat and/or A2RE RNAs encode proteins that could potentially affect calcium homeostasis and TMPyP4 can potentially affect multiple different pathways in the cell.

TMPyP4 binds to CGG repeat RNA and rescues granule translation and calcium homeostasis. The effect of TMPyP4 on calcium homeostasis may be secondary to the effect on granule translation if translation of RNAs that mediate calcium homeostasis is inhibited by CGG repeat RNA and rescued by TMPyP4. However, it is also possible that TMPyP4 affects calcium homeostasis directly. Previous studies have shown that TMPyP4 can induce calcium release by direct interaction with the Ca2+ release protein from sarcoplasmic reticulum [51]. Furthermore, studies in yeast suggest that TMPyP4 can induce a cellular oxidative stress response that could affect calcium homeostasis [52]. TMPyP4 rescue of calcium homeostasis in premutation fibroblasts could theoretically involve similar mechanisms.

The observation that granule translation and calcium homeostasis are dysregulated in premutation fibroblasts indicates that pathogenic effects of CGG repeat expansions in FMR1 (and possibly other CGG repeat genes) are manifested in non-neuronal cell types. In this study premutation fibroblasts that exhibit dysregulated granule translation and calcium homeostasis were obtained from apparently healthy young female premutation carriers with no apparent neurological or neuromuscular symptoms. This indicates that disrupted granule translation and calcium homeostasis in human fibroblasts may provide early preclinical biomarkers for identifying individuals at increased risk to develop late onset neurological or neuromuscular disorders such as FXTAS, FXPOI or other CGG repeat disorders. Chronic, sustained disruption of granule translation and/or calcium homeostasis may be deleterious to the cell over the long term and may contribute to late onset development of neurological or neuromuscular pathology in these individuals. Restoring normal granule translation and/or calcium homeostasis with TMPyP4 could potentially reduce the risk of subsequent onset of these disorders in premutation carriers. Inhibition of translation in granules by CGG repeat expansions in FMR1 RNA represents a potential pathogenic mechanism for CGG repeat expansion disorders such as FXTAS and FXPOI. Rescue of translation in granules by TMPyP4 could potentially ameliorate pathogenic effects in CGG repeat expansion disorders.
